# Effect of microporosity on scaffolds for bone tissue engineering

**DOI:** 10.1093/rb/rby001

**Published:** 2018-02-05

**Authors:** Ke Zhang, Yubo Fan, Nicholas Dunne, Xiaoming Li

**Affiliations:** 1 Key Laboratory for Biomechanics and Mechanobiology of Ministry of Education, School of Biological Science and Medical Engineering, Beihang University, Beijing 100083, China; 2 Beijing Advanced Innovation Center for Biomedical Engineering, Beihang University, Beijing 102402, China; 3 Centre for Medical Engineering Research, School of Mechanical and Manufacturing Engineering, Dublin City University, Stokes Building, Collins Avenue, Dublin 9, Ireland; 4 State Key Laboratory of New Ceramic and Fine Processing, Tsinghua University, Beijing 100084, China

**Keywords:** bone tissue engineering, mechanism, microporosity

## Abstract

Microporosity has a critical role in improving the osteogenesis of scaffolds for bone tissue engineering. Although the exact mechanism, by which it promotes new bone formation, is not well recognized yet, the related hypothesis can be found in many previous studies. This review presents those possible mechanisms about how the microporosity enhances the osteogenic-related functions of cells *in vitro* and the osteogenic activity of scaffolds *in vivo*. In summary, the increased specific surface areas by microporosity can offer more protein adsorption sites and accelerate the release of degradation products, which facilitate the interactions between scaffolds and cells. Meanwhile, the unique surface properties of microporous scaffolds have a considerable effect on the protein adsorption. Moreover, capillary force generated by the microporosity can improve the attachment of bone-related cells on the scaffolds surface, and even make the cells achieve penetration into the micropores smaller than them. This review also pays attention to the relationship between the biological and mechanical properties of microporous scaffolds. Although lots of achievements have been obtained, there is still a lot of work to do, some of which has been proposed in the conclusions and perspectives part.

## Introduction

Scaffolds for bone tissue engineering are attracting more and more attentions because they are heavily involved in regenerative medicine research [1, 2]. At present, numbers of synthetic alternatives with potential osteogenesis have been tried as artificial scaffolds, including ceramics, polymers, metals and composites [3–8]. However, material composition of scaffolds is not the only qualification to induce bone formation. In recent years, significant progress has been made toward porous scaffolds with desired osteogenesis [9–11]. Typically, macropororosity (pore size above 100 μm) is usually required to facilitate the osteogenesis and angiogenesis [12]. Interconnected macropores are necessary to promote body fluid circulation and cell migration to the core of the implant [13]. More importantly, people also found that microporosity (pore size smaller than 10 μm) plays a significant role in enhancing the osteoinduction of scaffolds [9, 10, 14, 15].

In fact, the cellular response to microporous scaffolds is a secondary event [10]. Although the exact mechanism, by which the microporosity promotes the osteogenic-related functions of cells and new bone formation, is still being debated, the related hypotheses can be found in many previous researches. Until now, several possible factors (e.g. proteins, degradation products and capillary force) have been found in the microporous scaffolds. The main possible related mechanisms can be summarized as follows.

Firstly, the presence of microporosity can significantly enhance the specific surface area and improve the permeability of scaffolds, thereby providing more protein adsorption sites and enhancing the degradation of scaffolds [16–18]. For one thing, the cells interact with microporous scaffolds by adsorbed more osteogenic-related proteins on their surface via the membrane receptor to achieve improved osteogenic-related functions (e.g. attachment, proliferation, differentiation, biomineralization, etc.) [9, 19, 20]. For another, the proper degradation rate of scaffolds in physiological environment is one of the essential characteristics for their applications in bone tissue repair and regeneration. Meanwhile, researchers have found that the degradation products, such as some ions (calcium (Ca), strontium (Sr), lithium (Li), magnesium (Mg), etc.), could have a significant impact on biocompatibility, osteogenesis and angiogenesis [21, 22]. Furthermore, an ideal scaffold requires its degradation rate consistent with the regeneration rate of new bone tissue [23]. Secondly, surface properties of scaffolds, such as surface roughness, surface free energy, surface charge, and chemical functionalities, also play a non-negligible role in the interactions between scaffolds and cells [19, 24, 25]. Particularly, surface roughness and surface free energy have a considerable influence on the protein adsorption [10, 19]. Thirdly, micropore-induced capillary force can not only anchor cells to the substrate surface but also deform the cells and draw them into the interconnect micropores, even if the micropore is smaller than cells [26, 27].

Indeed, the chemical composition of scaffolds is an important qualification for their osteogenic activities [6, 28, 29]. However, although with the same chemical composition, some scaffolds are osteoinductive, whereas others are not [30, 31]. The presence of microporosity has been shown to be of great importance for the osteoinduction of scaffolds [14, 32]. For example, microporous hydroxyapatite (HA) ceramics could induce bone formation after intramuscular implantation in dogs, whereas no new bone was formed in those lacking enough microporosity [33]. Therefore, the proper microporosity plays a positive role in the osteoconduction of scaffolds [34]. However, too high microporosity was shown to be detrimental to the mechanical properties of the scaffolds [35]. It is generally agreed that a perfect microporous scaffold should also possess satisfactory mechanical properties (e.g. stiffness, strength and toughness). Only if it possesses good mechanical properties, can the scaffold keep its shape and characters after being embedded in the body and provide the sufficient structural support during the bone formation [36–38].

This review firstly depicts the possible mechanisms about how the microporosity affects the osteogenic-related functions of cells *in vitro*. Then, many *in vivo* studies are reviewed to show the important role of microporosity in the osteoinduction and osteoconduction of scaffolds to make a connection with the above possible mechanisms. Finally, the pros and cons of microporosity in bone tissue engineering scaffolds are synthetically discussed from its effects on the biological performances and mechanical properties. Presenting the possible mechanisms about how the microporosity enhances the osteogenic-related functions of cells *in vitro* and the osteoconduction and osteoinduction of scaffolds *in vivo*, this review has the potential to not only arise more important related research points but also guide more scientific and reasonable design and preparation of bone tissue engineering scaffolds.

## Overview of the possible mechanisms about how the microporosity affects the osteogenic-related functions of cells *in vitro*

**Figure 1.**
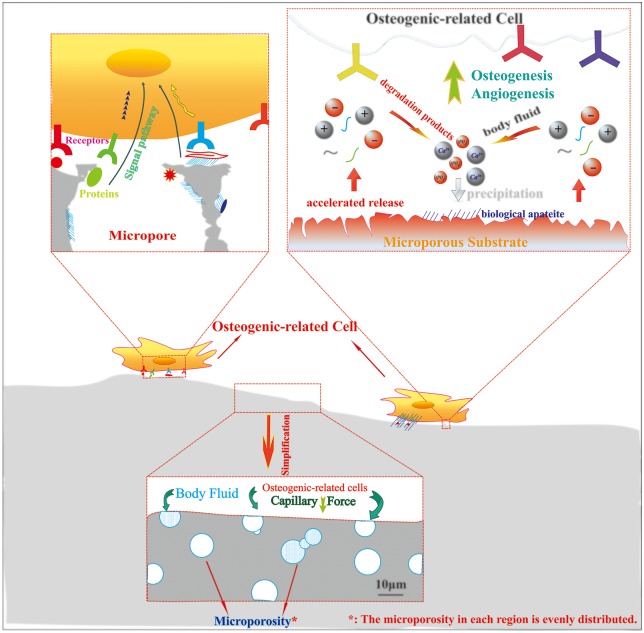
The mechanism diagram of scaffolds with microporosity affects the osteogenic-related cells *in vitro*

We have depicted the possible mechanisms about how the microporosity affects the osteogenic-related functions of cells *in vitro* in Fig. 1. The increased specific surface areas by microporosity can offer more protein adsorption sites and accelerate the release of degradation products, which facilitate the interactions between scaffolds and cells. Meanwhile, the unique surface properties of microporous scaffolds have a considerable influence on the protein adsorption. Moreover, capillary force generated by the microporosity can improve the attachment of bone-related cells on the scaffolds surface and even make the cells achieve penetration into the micropores smaller than them.

### Increased specific surface area by the presence of microporosity

#### Providing more protein adsorption sites

Numerous studies have shown that the specific surface area of scaffold could be enhanced with the increase of microporosity, thereby providing more protein adsorption sites. The adsorption sites are necessary for more proteins to be adsorbed on the scaffolds, and these proteins can subsequently stimulate the osteogenic-related functions of cells, such as attachment, proliferation, osteogenic differentiation and biomineralization [1, 9, 16, 39]. For example, Rouahi *et al.* [19] investigated the influence of the microstructure of microporous HA (the median of pore size is about 0.4 μm and open microporosity around 12%) on serum protein adsorption. Microporous HA adsorbed 10-fold more proteins, especially fibronectin and albumin, than non-microporous HA. Regarding the osteogenic differentiation, previous studies from our group have indicated that the microporous biphasic calcium-phosphate (BCP) scaffolds with larger specific surface area could concentrate more proteins (including bone-inducing proteins) that differentiate inducible cells, that murine myoblasts to osteogenic cells [9]. In this study, we prepared two types of BCP scaffolds (BCP1150-P and BCP1150-D), and the specific surface area of BCP1150-P was much higher than that of BCP1150-D, which is mainly attributed to the higher microporosity (48.4% *vs*. 24.3%). Then, murine myoblasts (C2C12) were incubated on the two kinds of scaffolds after immersion in recombinant human bone morphogenetic protein-2 (rhBMP-2) solution. The results showed that alkaline phosphatase (ALP)/DNA of C2C12 on BCP1150-P was significantly higher than on BCP1150-D at each culture time, which was probably because BCP1150-P, using larger surface areas, adsorbed more specific growth factors from culture medium than BCP1150-D, and the specific growth factors facilitate the differentiation of C2C12 into osteogenic cells. In addition, we also reported that by increasing microporosity and specific surface areas, the mineralization of human adipose-derived stem cells cultured on BCP-A (microporosity ≈ 50%) was more significant than on BCP-B (≈ 25%) at Day 7 [10]. However, many studies reported that higher microporosity was unfavorable for the proliferation of osteoblasts [19, 40, 41]. For example, Rosa *et al.* [41] demonstrated that rat bone marrow stromal cells (BMSCs) proliferation was greater on BCP scaffolds with microporosity rates of 5% and 15%, as compared with those with microporosity rate of 30%. Presence of proteins on scaffolds promoted bone cells attachment that directly affected the morphology of cells [42]. Particularly, cells appeared like ‘adsorbed’ by the HA surface and exhibited the particularity of the cytoplasmic edge undistinguishable from the surface, with only the extremity of the cells and lamellipodia visible [19]. In consequence of this higher attachment capacity, cell proliferation was decreased.

#### Accelerating scaffold degradation and their products influence bone formation

Generally, the presence of microporosity can significantly enhance the specific surface area and improve the permeability of scaffolds, thereby enhancing the degradation of scaffolds. Until now, some ionic degradation products of scaffolds have been found to play an important role in their osteogenesis and angiogenesis [43–48]. Besides, scaffolds combined with antibacterial properties would represent a promising solution to heal open fracture [49, 50]. For instance, copper-containing bioactive glass (BG) scaffolds could not only stimulate the expression of hypoxia-inducible factor-1α and vascular endothelial growth factor (VEGF) in human BMSCs but also significantly inhibit the viability of *Escherichia coli*, owing to the release of Cu ion [51]. Moreover, many studies have shown the effects of the released ions (e.g. Ca, P, Sr, Zn, B, Co, Li, Mg) from inorganic scaffolds, as summarized in Table 1. In fact, the effects of ions on the osteogenic-related functions of cells depend on not only their type but also their concentration. For example, Maeno *et al.* [62] found that low (2–4 mM) and medium (6–8 mM) Ca concentrations were, respectively, suitable for osteoblast proliferation, differentiation and biomineralization whereas higher Ca concentrations (>10 mM) are cytotoxic. Therefore, it is essential to obtain the optimum concentration of ions in extracellular matrix during the degradation process through designing the reasonable microporosity.

**Table 1.** Review the effect of released ions on biological response *in vivo*/*in vitro*

Material composition	Ions released	Role	Biological response *in vivo*/*in vitro*	References
CaP	Ca	Osteogenesis	Enhance the proliferation and the osteogenic-related factors (bone morphogenic protein-2 (BMP-2), osteocalcin (OCN), osteopontin (OPN), and bone sialoprotein (BSP)) expression of human bone marrow-derived mesenchymal stromal cells (MSC) MEK1/2 is involved in Ca^2+^ mediated the expression of BMP-2 in human MSC	[52]
42SiO_2_-4P_2_O_5_-37.05CaO-15Na_2_O-1.95SrO	Sr	Osteogenesis Antibacterial	ALP activity, cell number, type I collagen (Col I), and mineral nodule formation of MC3T3-E1 cells are significantly promoted Inhibit the growth of *Aggregatibacter actinomycetemcomitans* and *Porphyromonas gingivalis*	[53]
Srx-HA, *x* = Sr/(Ca + Sr) = 0, 10, 40, 100 mol.%	Sr	Osteogenesis Cytotoxicity	MG-63 cells attached and proliferated on the Sr10-HA and Sr40-HA surfaces No MG-63 cells are found on the Sr100-HA surface	[54]
Sr-Ca_2_ZnSi_2_O_7_	Ca Si Sr Zn	Osteogenesis	Increased the expression of osteogenic markers (Runx-2, OPN, OCN, and BSP) in human bone derived cell (HOB) Induced osteoconductivity in rat tibia defects	[55]
B-BG: *x*B_2_O3/15CaO/2.5P_2_O_5_/(82.5-*x*)SiO_2_ (*x*=0%, 5%, 10 mol.%)	B Ca Si P	Osteogenesis	ALP activity of BMSC have no obvious difference with different boron contents Enhanced the expression of Col I and Runx-2 of BMSC	[56]
Lithium-doped CaP	Li	Osteogenesis	Enhance the osteogenesis-related genes (Col I, bone gamma-carboxyglutamate protein (Bglap), osteoprotegerin (OPG), Runx2, β-catenin) expression of MC3T3-E1 Activate the Wnt/β-catenin pathway of MC3T3-E1 Increase new bone formation in rat tibial defects	[57]
ZnTCP/HA, Zn concentration (0, 0.32, 0.63, 0.88, and 1.26 wt. %)	Zn	Osteogenesis	Zinc concentration between 2.2 and 7.2 μg/ml stimulates osteogenic differentiation in both rat and human BMSC	[58]
Zinc-doped 45S5 BG	Zn	Cytotoxicity	High Zn ion concentration (122 μM) have cytotoxic effect on MG-63 cells	[59]
Cobalt BG/collagen	Co	Angiogenesis Osteogenesis	Enhance the expression of VEGF in Human umbilical vein endothelial cells (HUVEC) Upregulated the ALP activity of MC3T3-E1 cell Activates the hypoxia-inducible factor-1α pathway	[60]
Mg-*x*Cu (*x* = 0, 0.03, 0.19, 0.57 wt.%) alloys	Mg Cu	Osteogenesis Angiogenesis Antibacterial	Higher BMP-2, BSP and Col I expression Mg-0.03Cu exhibits the highest expression level of angiogenesis-related genes Mg-Cu alloy declines the number of *Staphylococcus aureus* colony	[61]

Polymer scaffolds have been widely studied for bone tissue engineering because of their satisfactory processing and mechanical properties [63]. However, some polymers are unfavorable for bone-related cells and new bone formation owing to their long degradation period and acidic by-product. To overcome these problems, many studies have successfully modulated the degradation rate of polymer scaffolds through appropriately designing microporosity [64]. Meanwhile, although numbers of researches have tried to add bioactive ceramics into polymers to neutralize the acidic by-products, the existing preparation processes and methods cannot manufacture the composite scaffolds with desired microporous structure [65, 66].

Moreover, some others insisted that the micropore could provide more nucleation sites for the bone-like biological apatite precipitation [67]. After microporous calcium phosphate (CaP)-based scaffolds are immersed into a physiological environment, the presence of microporosity can accelerate their degradation and the release of Ca^2+^ and ${\text{PO}}_{4}^{3}$^−^. Accelerated release of Ca^2+^ and ${\text{PO}}_{4}^{3}$^−^ makes their concentration to more easily reach the supersaturation levels in the surrounding environment, so that the biological apatite can more easily precipitate inside the scaffolds [15]. Furthermore, several studies have showed that even if the microporous scaffolds did not contain Ca or P, those ions from body fluid could also form biological apatites on their surface [68]. Therefore, the proper microporosity has a positive effect on the biomineralization of scaffolds. During this process, it was found that some proteins could easily coprecipitate with the biological apatite formation, which in turn triggered the differentiation of trapped cells toward the osteogenic lineage [69]. Although the exact family of protein contained in the formed biological apatites has not been determined, Benesch *et al.* [69] reported that they did include bone-associated non-collagenous proteins, which generally contained a long and negative charged sequence to form coordination bonds with Ca^2+^ ion in biological apatite.

### Surface properties of microporous scaffolds

Surface properties of scaffolds appeared to play a dominant role in the interactions between scaffolds and bone-related cells [70–72]. And, it is well recognized that the microporosity has a significant effect on surface properties. The representative surface properties, the surface roughness and surface free energy of microporous scaffolds have been regarded as two important factors to regulate the cellular functions [71, 73, 74].

Numerous researchers have reported that the osteogenic-related functions of cells were influenced by surface roughness. For example, Itälä *et al.* [75] reported that the microroughened surface of BG enhanced the attachment of human osteoblast-like cells (MG-63) but did not have a significant effect on the proliferation as compared to the smooth surface. Osteoblastic differentiation of human MSCs was increased on the Ti surfaces with micron-scale rough (Ra = 4 μm) [76].

Furthermore, many *in vitro* studies have been launched to find out how different surface roughness affected the osteogenic-related functions of cultured cells, but their results were not consistent [77, 78]. For example, Kunzler *et al.* [79] showed that the proliferation of rat calvarial osteoblasts on Ti surface was improved with the increase of the surface roughness in the range of 0–4 μm Ra value. Osteogenic differentiation of bone marrow mesenchymal stem cells was promoted by polycaprolactone surfaces with the Ra values of about 2–3 μm [78]. However, Andrukhov *et al.* found that MG-63 osteoblasts proliferation was decreased with increasing surface roughness after culturing the cells on Ti surfaces with different micrometer-scale surface roughness (0, 1, 2 and 4 μm). Meanwhile, the expression of ALP, osteocalcin (OCN) and VEGF on the surfaces with the Ra values of 1 and 2 μm was shown significantly higher than that on the other two groups [80].

To optimize surface roughness by appropriately controlling the microporosity, we need to understand its influencing mechanism. Although the exact reasons, by which surface roughness affects the osteogenic-related functions of cells, are not well recognized yet, the related hypothesis can be found in previous studies. For instance, Deng *et al.* [81] found that the cell attachment on the microrough surface was better than that on the polished one due to more binding sites for proteins. In addition, previous studies have reported that microroughened titanium substrates induce osteoblast differentiation and inhibit osteoclast activity by stimulating some specific integrin signaling, such as α2β1 [76, 82, 83].

As for surface free energy, it is directly related with surface wettability. Normally, lower surface free energy corresponds to lower hydrophilicity. It is well known that surface wettability is one of important influencing factors on protein adsorption. For example, Kennedy *et al.* [84] reported that the cell proliferation was increased on the hydrophobic surface because of more fibronectin adsorbed. Therefore, protein adsorption is one of the main means for surface free energy to affect cellular functions. For example, Fang *et al.* showed that MG63 cells cultured on the titanium surface with high free energy showed an enhanced production of OCN and osteoprotegerin (OPG), phospholipase D mRNA and activity, and ALP activities because of protein adsorption. They also found that the osteogenic response was induced via protein kinase C-dependent signaling [85].

Above all, we can see that it is very important to obtain desired surface properties by effectively controlling microporosity of bone tissue engineering scaffolds.

### Capillarity

**Figure 2.**
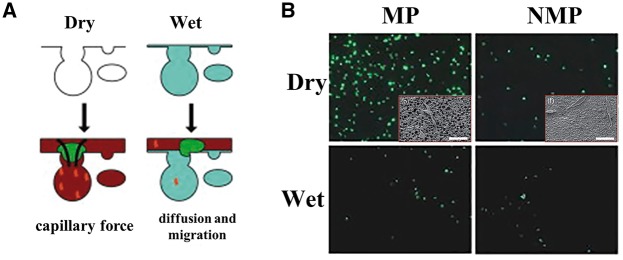
Capillary force in microporous substrate for the cell attachment and penetration. (**A**) Schematic of cell attachment to the dry and wet microporous substrates, respectively. Dry microporous substrates with capillary force will actively draw in cells and proteins in solution. MP substrate with PBS-filled pores must rely on migration and diffusion for cells and proteins to localize. (**B**) Fluorescent imaging shows the localization of D1 cells on microporous and non-microporous substrates for the dry and wet (PBS-filled) substrates, respectively. Insert pictures are SEM images of the dry microporous and non-microporous substrates surface with D1 cells. Scale bars = 50 μm [27]

Several studies demonstrated that there were improved outcomes in repair of large bone defects when seeding osteogenic-related cells into microporous substrates before implantation, but the physics underlying cellular attachment on such scaffolds remains elusive [86]. In recent years, several studies tried to confirm the hypothesis that microstructure-induced capillary force could enhance the cell attachment on the scaffolds surface [27]. Polark *et al.* [27] reported that the dried microporous substrate could facilitate the attachment of cells on their surface through generating capillary force, whereas the phosphate-buffered saline (PBS)-filled (wet) microporous substrate relies on the diffusion and migration of cells and proteins, as shown in Fig. 2A. They hypothesized that capillary force was generated by dry microporous substrates but not wet microporous substrates or substrates without micropores. Similarly, the study of Bai *et al.* [87] showed that the MSCs could be self-loaded under capillary flow into dry scaffolds while the cells could not attach on the scaffolds pre-perfused with PBS but only on the surface of petri dish, due to the lack of capillaries. Fluorescent images showed that dry microporous substrates had the greatest cell density, and there was no difference between the non-microporous and wet microporous substrates (Fig. 2B). Scanning electron microscopy (SEM) images of dry microporous and non-microporous substrate surface with D1 cells suggested that the cells attached much better on dry microporous surface due to the capillary force (Fig. 2B). Furthermore, Polark *et al.* [27] reported that the cell penetration depth within microporous substrates *in vitro* depended on the cell type and the capillary forces generated by micropores. The critical pressure for the cell to enter a micropore is lower, cell penetrated more easily into micropores [27]. This work clearly demonstrated that capillary forces played an important role in cell attachment on the dry microporous substrate surface, and these forces could deform the cells and draw them into micropores.

## Improving the osteogenic activity of scaffolds *in vivo*

Currently, besides the *in vitro* studies, many researchers have investigated into the microporous scaffolds by animal experiments *in vivo* to find out their potentials as bone tissue engineering scaffolds, providing more direct data for their possible further clinical applications. As described above, *in vitro* studies showed that the microporosity played an important role in the osteogenic-related functions of cells; therefore, the effects of microporosity on the osteogenic activity of scaffolds *in vivo* (e.g. osteoinduction and osteoconduction) deserve further study.

### Osteoinduction

Recently, scaffolds with microporosity have been reported to be osteoinductive in several animal models after heterotopic/ectopic implantation (e.g. intramuscularly or subcutaneously). For example, Habibovic *et al.* [32] prepared two types of calcium phosphate ceramics: HA and BCP by sintering and determined the role of microporosity in their osteoinduction. Sintering temperatures between 1100 and 1200°C only modified the microporosity (pore diameter < 10 μm), and the microporosity was significantly decreased with increasing sintering temperature. They implanted the samples into the back muscles of Dutch milk goats and harvested at 6 and 12 weeks post-implantation. Histomorphometry analysis showed that new bone was formed in the all samples except HA1250 (the lowest microporosity). They pointed out that the specific surface area of scaffolds increased with the increase of microporosity, and hence accelerated the release of ions. The higher ion concentration facilitated the formation of apatite *in vivo*, causing the coprecipitation of the osteogenic-related proteins (e.g. BMPs) that had in turn simulated the differentiation of cells toward the osteogenic lineage [32]. Similarly, Chan *et al.* [3] determined the relationship between increased microporosity and the osteoinductive potential of silicate substituted calcium phosphate (SiCaP) bone substitutes in an *in vivo* ectopic model. They respectively implanted SiCaP scaffolds with different microporosity (22.5%, 32.0% and 46.0%) into the ovine parapsinalis muscle [3]. Histomorphometry analyses showed that the SiCaP-46 group had larger amount of new bone formation (6.17 ± 1.51%) than the SiCaP-22.5 group (1.33 ± 0.84%) at 12 weeks after operation, and that bone formation was observed in pore size smaller than 10 μm [3].

In addition, dog is another commonly used animal experiment subject to test the osteogenic activity of scaffolds [88]. For instance, Zhang *et al.* reported that microporous CaP ceramics might induce ectopic osteogenesis through surface architecture. The two kinds of ceramics were only different in their surface architecture, BCP-R had a surface roughness of 325.4 ± 58.9 nm whereas BCP-S was 231.6 ± 35.7 nm, which were respectively implanted into the paraspinal muscle of dogs for 12 weeks [89]. The result showed that more bone was formed in BCP-R than in BCP-S.

Besides, several studies have also evaluated the role of microporosity in the osteoinduction of microporous scaffolds by subcutaneous implantation. For example, Dorj *et al.* [90] prepared microporous PLLA scaffold using room temperature ionic liquid as the counter-interpenetrating phase and pore generator. The scaffolds possessed about 70% microporosity with average pore size of 2.43 μm. They respectively implanted the microporous and non-microporous scaffolds into the back subcutaneous tissues of male Sprague–Dawley rats. The results showed that the microporous scaffolds possessed better tissue compatibility and could favor new bone formation better than the non-microporous scaffolds.

### Osteoconduction

Osteoconduction refers to bone ingrowth from bone defect edges toward the surface or down into the pores of scaffolds [42, 91]. It has been reported that the presence of microporosity could improve the bioactive of scaffolds and facilitate the new bone formation [92]. Previous studies have explored the micropore as an additional and important space for bone ingrowth [93, 94]. For instance, Campion *et al.* [94] implanted microporous SiCaP with different microporosity (23%, 32% and 46%) into the distal femur defect of adult ewes. Their results showed that scaffolds with higher microporosity (32% and 46%) displayed higher new bone formation after 8 weeks. They speculated that the permeability of scaffolds was increased with the increasing microporosity, thereby causing increased physical accessibility of nutrients to support the osteogenic-related functions of cells. Moreover, the higher microporosity corresponds with the higher specific surface area, which could promote the osteogenic-related proteins adsorption, thus subsequently lead to more new bone formation [94].

Some others have also investigated the potential of capillary force in improving the osteoconduction of microporous scaffolds *in vivo*. Polak *et al.* [27] implanted the microporous scaffolds into the pig mandibles defect, and the results showed that the endogenous cells were drawn into the micropores via capillary force. In a recent publication, Rustom *et al.* [95] demonstrated that micropore-induced capillary forces enhanced the homogeneity of bone distribution in scaffolds after the implantation into porcine mandibular cylindrical defects (8 mm in diameter). The results of heatmap and bone growth front contour showed that the bone growth front extends closer to the center in microporous-dry scaffolds than in microporous-wet and non-microporous scaffolds, and bone distribution was more homogenous in microporous-dry group than in the other groups. These results demonstrated that micropore-inducted capillarity enhanced the bone growth into scaffolds through the enhancement of bone distribution [95]. Therefore, the carefully design of microporosity in scaffold can be used to direct or drive the blood and marrow components into the scaffold through capillarity, which may lead to more effective treatment of the large bone defects.

Besides, some studies demonstrated the effectiveness of microporous scaffolds for the healing of osteochondral defects. For example, Bernstein *et al.* [96] seeded autologous chondrocytes into their prepared microporous calcium phosphate ceramic *in vitro*, and then implanted the composites into the medial femoral condyle of ovine knees osteochondral defects. The results showed that the microporous scaffolds could promote early bone ingrowth better. Moreover, the degradation of the scaffolds consisted with new bone formation, meanwhile the microporous β-tricalcium phosphate (TCP) scaffold had sufficient mechanical strength to support functional loading.

## The relationship between the biological and mechanical properties of microporous scaffolds

Philosophy tells us that everything has properties of two sides, which seems to be shown in the microporosity. Ideal scaffolds for bone tissue engineering should be biocompatible, osteoconductive, osteoinductive and have appropriate mechanical properties [97]. Although the microporosity plays an important role in promoting the osteogenic-related functions of cells and bone formation, it normally has a negative effect on the mechanical properties. For instance, Eqtesadi *et al.* [98] studied the effect of microporosity on the biological mechanical properties of 13–93 BG scaffolds. They immersed the scaffolds into stimulated body fluid to mimic *in vivo* degradation behavior and found that the formed HA crystal on the microporous scaffold surface was larger and more numerous than that on the dense scaffold surface. However, the presence of the microporosity significantly reduced their mechanical strength [98].

Many studies reported that the permeability of scaffolds was increased with increasing microporosity, which helpful for the nutrient transfer, thereby enhancing their osteoconduction [99, 100]. It is interesting that the new bone tissue can fill into the void space of the microporous scaffold and then form porous material-bone composite, thereby improving the mechanical properties of scaffold [101].

The mechanical properties of microporous scaffold are strongly influenced by microporosity and should be carefully designed. Until now, several strategies have been used to fabricate 3D microporous scaffolds in the past decades, including gas foaming technique [102], porogen-leaching process (e.g. salt or sucrose) [103, 104], freeze casting methods [105], rapid prototyping or solid freeform fabrication [100, 101], and others. Table 2 shows the fabrication technique of the scaffolds with microporosity for bone tissue engineering. However, they either have relatively complex processing technique or cannot control micropore size and microporosity accurately. For example, hierarchically 3D microporous/macroporous magnesium-calcium phosphate (micro/ma-MCP) scaffolds were fabricated from cement utilizing leaching method in the presence of sodium chloride (NaCl) particles and NaCl saturated water solution [108]. In this system, NaCl particles produced macropore, while NaCl solution acted as both cement liquid and porogens, inducing the formation of micropore. From the results of SEM, the highly interconnected micropores (pore sizes around 2–5 μm) were distributed across the macropore walls, and the morphology of micropores was irregular shape. The compression strength of micro/ma-MCP scaffolds (8.62 MPa) was close to that of cancellous bone. When the scaffolds were immersed into Tris–HCl solution, the Ca, Mg and P ion concentrations of micro/ma-MCP scaffold were increased faster than those of ma-MCP scaffold. Furthermore, the micro/ma-MCP scaffold could promote the osteogenic-related functions of MG63 cell (attachment, proliferation and ALP active) as compared to the ma-MCP scaffold [108].

**Table 2.** The fabrication technique of the scaffolds with microporosity for bone tissue engineering

Component	Technique	Pore characteristic	*In vivo/In vitro*	Mechanical properties	Result	References
13-93 BG	Robocasting	Multi-scale porosity	*In vitro* C2C12	The compressive strength (114±24 MPa) within the range of human cortical bone	The surface of scaffolds was fully covered by a HA layer after immersion in SBF for 7 days Cells thoroughly cover the surface of scaffold after 16 days	[98]
Porous titanium	Centrifugal granulation technology and stack sintering	Macropores (180.0–341.8 μm) and micropores (6.1–11.8 μm)	*In vitro* MSCs	The compressive strength of the scaffolds (83.4–108.9 MPa) was high enough for the repair of load-bearing bone defects	Promoted the growth of cells	[106]
TCP/alginate	Rapid prototyping	Macro and microporosity	*In vitro* human osteoblast cells	The compressive strength of 60/40 sample around 20 MPa higher than human trabecular bone (0.5–15 MPa)	Allowed cells anchoring and proliferation at scaffold surface	[107]
HAP/45S5 bioglass	Polyurethane foam templates	Macropores of 210–1100 μm with microporosity of 1–10 μm	*In vitro* MG63 cells	Compressive yield strength (0.8 MPa) close to the upper range of cancellous bone	Cells were successfully seeded on the scaffold surface	[105]
Polycaprolactone	Freeze extraction process	Microporosity in the walls of a macropore	*In vitro*	Permeability decreases with reduced microporosity	The microporous scaffold has potential used for cartilage regeneration	[100]
HA	Directed deposition technique	Micropores size range from 2 to 8 μm	*In vivo* the latissimus dorsi muscle of pig	The compressive stiffness of implanted scaffold (1.11±0.8 GPa) was less than that of human trabecular bone	The MP scaffolds contained bone after 8 weeks	[38]

As described above, the osteogenic activity of scaffolds is improved with increasing of microporosity, whereas their mechanical property is decreased. Therefore, it is very important to strike a balance between the mechanical and biological properties of scaffolds through rationally designing the microporosity, the existing manufacturing process or research means cannot achieve that it is important to balance the biological and mechanical properties of scaffolds, which can be very helpful to furthest expand their applications.

## Conclusions and perspectives

This review compiles the possible mechanisms about the role of microporosity in bone tissue engineering scaffolds. As mentioned in this article, the increased specific surface areas by microporosity can offer more protein adsorption sites, by which the cells have more opportunities to interact with the osteogenic-related proteins, and therefore facilitate the cellular osteogenic functions to form new bone tissue. Meanwhile, the high specific surface areas can also accelerate the release of degradation products to improve the osteogenesis, the mechanism of which may be involved in the two aspects as follows. Some ionic products have an irritant effect on the osteogenic-related functions of cells. In addition, microporous scaffolds containing calcium phosphate can accelerate the supersaturation of Ca^2+^ and ${\text{PO}}_{4}^{3}$^−^ in the micropores vicinity, thereby promoting the biological apatite to precipitate inside the scaffolds. Besides, the unique surface properties of the microporous scaffolds, particularly surface roughness and surface free energy, also have considerable effect on the protein adsorption. Moreover, capillary force generated by the microporosity can improve the attachment of bone-related cells on the scaffolds surface, and even make the cells achieve penetration into the micropores smaller than them.

Although many studies on microporosity for bone tissue engineering have been done and many achievements have been gotten, there is still a lot of work to do. Only once we have a better understanding of the mechanisms about how microporosity interacts with biological molecules, cells and bone tissue, can we design more efficient scaffolds for bone tissue engineering.

Firstly, it is well recognized that mechanical properties are very important for scaffolds in bone tissue engineering applications, and that the uniformity of the scaffold structure affects its mechanical properties. However, from most of the current studies, we can see that micropores in the prepared scaffolds are random and heterogeneous. Therefore, the novel techniques and methods need to be developed to prepare more homogeneous micropore structure.

Secondly, as the interactions between proteins and substrates are largely dependent on the substrate structures, the difference of micropore structure has a significant influence on the type and amount of the adsorbed proteins, which thereafter affect the osteogenic-related functions of the surrounding related cells and formed new bone quality and quantity. However, it is still unclear how micropores with specific structures affect the adsorption of specific proteins. Therefore, more meticulous and deeper researches into the interrelationship between the specific micropore structures, the type and amount of the adsorbed proteins, and cell signaling pathways related to osteogenic-related functions are necessary to guide more scientifically the design and fabrication of the scaffolds with desired micropore functions for bone tissue engineering.

Moreover, with the development of early bone formation and the biodegradation of scaffolds, the structure of micropores changes, which directly leads to the alteration of the type and number of bioactive molecules that interacts with scaffolds, thereby affecting the real-time biofunctions of the microporous scaffolds. So, it is very necessary to investigate into the change law and mechanism of the micropore structure in specific bone tissue engineering scaffolds *in vitro* and *in vivo* by computer simulation and real experiments to find out the main own influencing factors, and more scientifically design and prepare the microporous scaffolds. Furthermore, the interaction style between scaffolds and proteins also changed, which, however, few (if any) studies have reported either. Therefore, much deeper related investigations are necessary, so that more desired specific proteins can be adsorbed during different stage of biodegradation and new bone formation, thereby reducing the time required for defects healing.
